# Evaluation of salivary parameters and dental status in adult hemodialysis patients in an indian population

**DOI:** 10.4317/jced.54633

**Published:** 2018-05-01

**Authors:** Preethesh Shetty, Mithra N. Hegde, Sunil M. Eraly

**Affiliations:** 1 MDS [Conservative Dentistry & Endodontics], A.B.Shetty Memorial Institute Of Dental Sciences, Nitte University, Deralakatte, Mangalore-575018; 2 MDS [Conservative Dentistry & Endodontics], Ph.D, MAMS, A.B.Shetty Memorial Institute Of Dental Sciences, Nitte University, Deralakatte, Mangalore-575018; 3 MDS [Conservative Dentistry & Endodontics], Malabar Dental College, KUHAS University, Kerala

## Abstract

**Background:**

Renal failure is a process that expresses a loss of functional capacity of the nephrons, independently of its etiology. The most widely used technique to combat renal failure is hemodialysis. Renal failure causes various systemic alterations including oral complications such as variations in the flow and composition of the saliva. Caries is a multifactorial disease and impaired stimulated salivary flow rate and buffering capacity are the best-known risk factors. The present study aims to evaluate the salivary pH, buffering capacity and the flow rate of saliva to the DMFT status in adult hemodialysis patients among the Indian population.

**Material and Methods:**

Twenty healthy individuals and sixty patients undergoing hemodialysis were divided into four groups based on the following criteria: Group 1: Control group; healthy individuals,Group 2: Patients before undergoing dialysis or undergoing dialysis<3 months, Group 3: Patients undergoing dialysis since 6 months-2 years, Group 4: Patients undergoing dialysis>2 years. Dental examinations were performed according to the modified WHO oral health survey 2013 criteria and DMFT index. Saliva was collected after pre-stimulation to measuring the flow rate, buffering capacity and pH.

**Results:**

The results exhibited a decrease in the salivary flow rate and buffering capacity with the increase in the time interval of hemodialysis, but salivary pH was found to be increasing with time. A direct relationship was seen between the DMFT scores with the increasing time interval. There was a significant correlation between DMFT index, stimulated salivary flow rate, and buffering capacity in the patients.

**Conclusions:**

Oral health impairment can beacon to grave problems in infection-prone hemodialysis patients. Hence, the patients on hemodialysis should have regular dental examinations and treatment. Regular dental examination and instruction in patients awaiting a renal transplantation is of vital importance to ensure optimal oral health.

** Key words:**Saliva, pH, Buffering Capacity, Flow Rate, Hemodialysis, DMFT.

## Introduction

Saliva is a clinically ostensive biofluid efficacious for novel conceptualization to prognosis of a disease, laboratory diagnosis, clinical diagnosis, monitoring of diseases and treatment of patients of both oral and systemic origin (1). Saliva is known to play a highly important role in maintenance of the oral health (2). Empirical to the elements of saliva, it is known to possess properties such as digestion, lubrication, neutralization, clearance of unwanted products, remineralisation and antimicrobial activity (3). The flow rate, buffering capacity, calcium ion, phosphate ion and fluoride ion concentrations are indispensible for protection against caries (4).

Dentists encounter various systemic diseases in clinic practice, one such being chronic renal failure or end-stage renal disease. Over the last few decades, the prevalence and incidence of patients with end-stage renal disease has been increasing (5). Hemodialysis remains the standard therapeutic intervention. The procedure involves an artificial simulation of the renal system by detoxification of nitrogen and other metabolic products from the blood by using a hemodyalizing system (6). The oral manifestations in these patients may be due to numerous factors, such as relative state of immunosuppression, renal osteodystrophy, medications, restriction of oral fluid intake and bone loss (7,8).

The risk of oral infections may also aggravate the risk of septicemia and endocarditis in dialysis patients. The condition has been noted to be exacerbated by ill-fitting oral prosthesis, caries and local infections (9). Therefore, assuring a healthy dentition is highly fundamental in patients who are candidates for renal transplantation due to the immunosuppressive protocols, which may further predispose to oral and possibly disseminated infection (10).

Dental caries has been advocated to be a multifactorial disease, where impaired stimulated salivary flow rate and buffering capacity are noted as the best-known risk factors (5). Hence , the present study aims to evaluate the salivary pH, buffering capacity and the flow rate of saliva to the status of the dentition as recorded by the modified WHO oral health survey 2013 in adult hemodialysis patients among the Indian population.

## Material and Methods

The present study was conducted on a total of 80 individuals; 20 healthy individuals and 60 patients undergoing hemodialysis treatment in the Department of Nephrology at K.S Hegde Hospital. Ethical clearance was acquired from the Institution Ethics Committee. Patients under the age of 18 years and complete edentulous patients were excluded from the study.

The patients were divided into four groups based on the following criteria:

Group I: Control group [ 20 patients ] – healthy individuals., Group II: Patients undergoing dialysis < 3 months [20 patients].Group III: Patients undergoing dialysis from 6 months-2 years [20 patients] and Group IV: Patients undergoing dialysis > 2 years [20 patients].

After obtaining the patient`s informed consent, the dental examinations were performed according to modified WHO oral health survey 2013 criteria and DMFT index. The teeth were isolated with cotton rolls & examined using a mouth mirror & probe under good illumination.

All the individuals taking part in the study were asked to abstain from smoking, drinking, eating and performing oral hygiene procedures, 2 hours before the collection of saliva samples (11). The sample collection was done for nearly 5 min, between 9:00 a.m. and 11:00 a.m. The analysis of the saliva samples was conducted by using the saliva-check kit (GC Asia Dental Pvt. Ltd., Singapore, 508724). All samples were collected post the hemodialysis procedure, at the nephrology department.

-Flow rate (12)

The saliva was collected into a collection cup after pre-stimulation. Whole saliva was stimulated with the aid of paraffin-wax chewing method. The saliva was collected from each subject for 5 min. For the measurement of flow rate, the collection of saliva into the collection cup was timed. It was expressed as ml/min. it was evaluated based on the following readings as per the saliva check kit: <3.5ml- very low, 3.5-5.0ml-low, >5.0ml-normal.

- Buffering capacity (12)

Sufficient saliva was drawn from the collection cup using a pipette, followed by a drop of saliva each being dispensed onto the three test pads. After 2 minutes, the test pads started to change color.

Depending upon the final color on the test pad, the total number of points was calculated. The combined total buffering capacity of saliva was interpreted based on the saliva buffer capacity indicator available with the kit: green – 4, green/blue-3, blue-2, red/blue-1, red-0. Depending upon the total points, the buffering ability of the saliva was deciphered based on the following scores; 0-5:very low, 6-9:low, 10-12: normal/high.

-pH measurement (12)

The saliva was pipetted onto a pH test strip for 10 seconds. The color obtained on the test strip was compared against the salivary pH indicator. The salivary pH indicator is a testing chart available in the package.

The data obtained was statistically analyzed Kruskal-Wallis Test and Mann -Whitney U Test.

## Results

An inverse relationship was observed between the decrease in the flow rate of saliva in hemodialysis patients to the increasing time intervals. Pateints under dialysis for more than 2 years had the least mean (SD) salivary flow rate of 3.63 (1.32) followed by patients under dialysis for 6 months to 2 years (4.95±1.60), under dialysis for < 3months (6.55±1.69), and highest in control group (9.20±1.70). The median salivary flow rate in those subjects is 3.25, 4, 5.80 and 9 respectively. There was a significant difference in the distribution of the salivary volume among the study groups (*p*<0.001). On pairwise comparison between the study groups, the difference in the distribution of salivary volume was found to be statistically significant between all the groups (<0.05) ( Table 1).

**Table 1:**
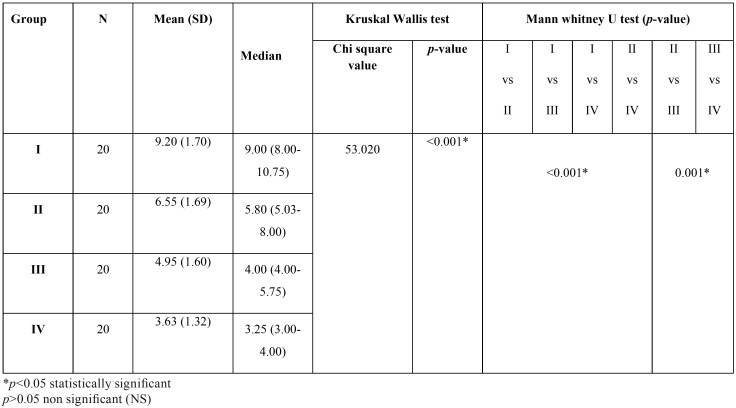
Comparison of the variation of the flow rate of saliva in adult hemodialysis patients compared to healthy controls at different time intervals of treatment.

An inverse relationship was observed between the decrease in the buffering capacity of saliva in hemodialysis patients to the increasing time intervals. Patients under dialysis for more than 2 years had the least mean (SD) salivary buffering capacity of 3.65 (1.84) followed by patients under dialysis for 6 months to 2 years (7.50±2.26), under dialysis for < 3months (8.80±2.09), and highest in control group (10.95±1.19). The median buffering capacity in those subjects is 4, 8, 10 and 11.50 respectively. Patients under dialysis for more than 2 years had the least mean (SD) salivary buffering capacity of 3.65 (1.84) followed by patients under dialysis for 6 months to 2 years (7.50±2.26), under dialysis for < 3months (8.80±2.09), and highest in control group (10.95±1.19) ( Table 2).

**Table 2:**
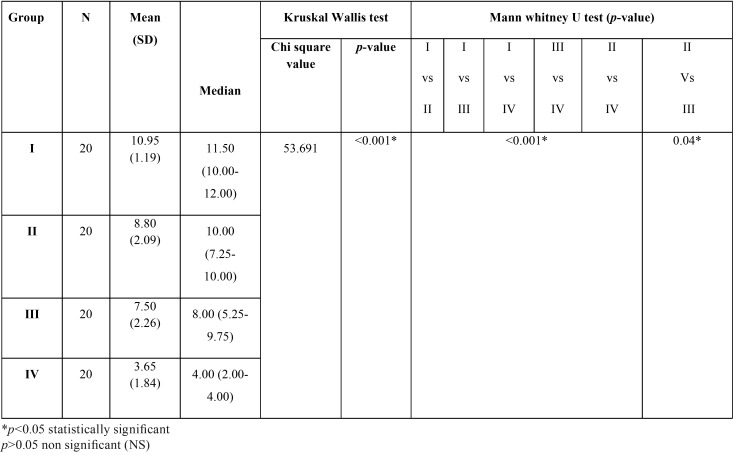
Comparison of the variation in the buffering capacity of saliva in adult hemodialysis patients compared to healthy controls at different time intervals of treatment.

A direct relationship was observed between the increase in the pH of saliva in hemodialysis patients to the increasing time intervals. Patients under dialysis for more than 2 years had the highest mean (SD) salivary pH of 7.33(0.35) followed by patients under dialysis for 6 months to 2 years (6.75 ±0.35), under dialysis for < 3months(6.38±0.32), and lowest in control group (6.02±0.27). The median pH in those subjects is 7.45,6.80.6.25 and 6 respectively. There was a significant difference in the distribution of the pH levels among the study groups (*p*<0.001). On pairwise comparison between the study groups, the difference in the distribution of pH levels was found to be statistically significant between all the groups (<0.05) ( Table 3).

**Table 3:**
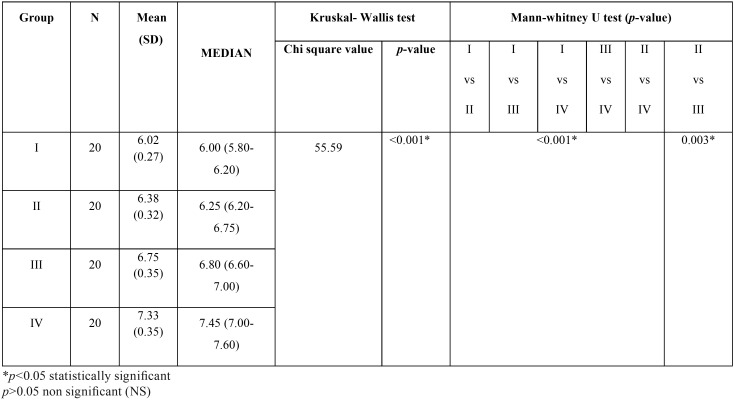
Comparison of the variation in the ph of saliva in adult hemodialysis patients compared to healthy controls at different time intervals of treatment.

A direct relationship was observed between the increase in the DMFT scores in hemodialysis patients to the increasing time intervals. Patients under dialysis for more than 2 years had the highest mean (SD) DMFT score of 16.85 (4.58) followed by patients under dialysis for 6 months to 2 years (12.65±3.33), under dialysis for < 3months (8.90±3.08), and least in control group (3.00±2.15). The median DMFT score in those subjects 15.50, 12.50, 8 and 2.50 respectively. There was a significant difference in the distribution of the DMFT score among the study groups (*p*<0.001) On pairwise comparison between the study groups, the difference in the distribution of DMFT score was found to be statistically significant between all the groups (<0.05) ( Table 4).

**Table 4:**
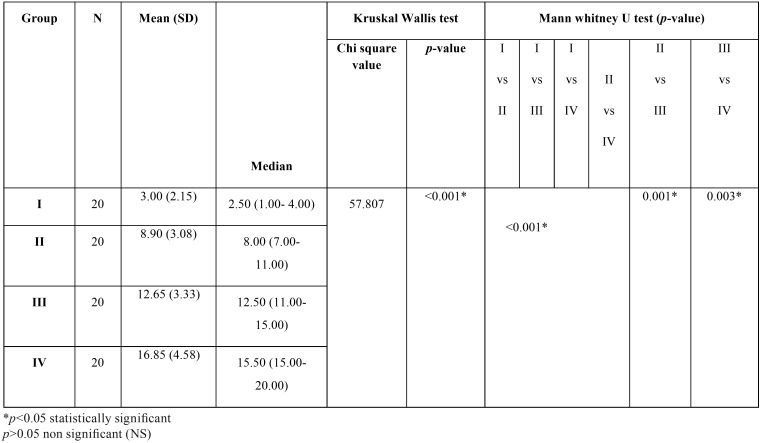
Comparison of the dmft values in adult hemodialysis patients compared to healthy controls at different time intervals of treatment.

## Discussion

Earlier research has reported deteriorating oral status of patients undergoing hemodialysis in comparison to that of the general population (13,14). Hence, the present study aimed to evaluate the salivary pH, buffering capacity and the flow rate of saliva and the status of the dentition as recorded by the DMFT index in adult hemodialysis patients in India.

-Evaluation of pH & Buffering capacity

In the present study, there existed a direct relationship between the increase in the salivary concentration of pH with increasing time intervals whereas an indirect relationship was noted between the decrease in buffering capacity of saliva with increasing time intervals. This is an invariable finding in all the studies that have evaluated these salivary parameters, both in adults as well as in children (15-17). The increase in the salivary pH could be attributed to the increase in the salivary urea levels (18-20). Urea acts through a double mechanism; firstly, bacterial urease metabolizes urea to carbon dioxide and ammonium ion causing an alkalinizing effect; and secondly, urea generates a periphrastic response of plaque in metabolizing carbohydrates to acid catabolites (21). It is quantized that the production of hydrogen ions drops down by tenfold in dialysis patients (22). This can be ascribed to the lowering buffer capacity of saliva with increasing time intervals of hemodialysis. Hence, urea plays an essential role in the alkalization and buffering of saliva.

-Evaluation of salivary volume

In the present study, an inverse relationship between the flow of saliva with the increase in the time interval of hemodialysis treatment was noted. Decrease in the salivary flow in patients undergoing hemodialysis has been reported; reaching levels lower than the limits of hypo salivation (8,23). It has been hypothesized that this may be due to direct damage to the glands and refrainment of fluid intake in patients undergoing hemodialysis treatment (7,19). However, these results are in contrast with the evaluation by Bots *et al.*, who advocated that the salivary flow in dialysis patients remained within the normal range (8).

-Evaluation of dmft index

In the present study, there was an inverse relationship between DMFT scores with the increase in the time interval of hemodialysis treatment. This can be attributed to the decreased salivary flow among the individuals along with decreased buffering capacity (8,15). Although the pH was increased, the increasing DMFT scores in the present study could be due to poor oral hygiene and poor maintenance, food impaction due to periodontitis, which provided an ideal environment for caries formation. The total DMFT score demonstrated a significant difference between the groups in our study (*p* <0.05).

Research has advocated that in patients undergoing hemodialysis, oral home care effectuation tends to be less prevalent when they did seek dental care on a regular basis (24,25).

In the present study, the salivary collection and analysis was done post hemodialysis. The limitations of this study could be that it has to be further expanded by increasing the number of samples and checking the salivary parameters pre and post hemodialysis procedures at different time intervals.

## Conclusions

The present study concluded that the salivary parameters flow rate and buffering capacity decreased with increasing time intervals whereas pH increased with increasing time intervals. Owing to these results, the DMFT scores were also found to be increasing with the passage of time. Accordingly, monitoring of pH, buffering capacity and volume should certainly be considered as a part of the routine sialometric assessment in renal transplant and dialysis patients. Oral infections and manifestations can lead to serious problems in dialysis patients who are prone to infections. Therefore, regular dental examinations and treatment should be made a fundamental protocol in these patients. Future studies in much larger cohorts of transplant and dialysis patients at different time interval should be conducted to evaluate the efficacy and feasibility of salivary analysis.
